# Mitochondrial Quality Control and Muscle Mass Maintenance

**DOI:** 10.3389/fphys.2015.00422

**Published:** 2016-01-12

**Authors:** Vanina Romanello, Marco Sandri

**Affiliations:** ^1^Venetian Institute of Molecular MedicinePadova, Italy; ^2^Department of Biomedical Science, University of PadovaPadova, Italy; ^3^Institute of Neuroscience, Consiglio Nazionale delle RicerchePadova, Italy; ^4^Department of Medicine, McGill UniversityMontreal, QC, Canada

**Keywords:** atrophy, mitochondria, fission, fusion, biogenesis, autophagy, muscle, sarcopenia

## Abstract

Loss of muscle mass and force occurs in many diseases such as disuse/inactivity, diabetes, cancer, renal, and cardiac failure and in aging-sarcopenia. In these catabolic conditions the mitochondrial content, morphology and function are greatly affected. The changes of mitochondrial network influence the production of reactive oxygen species (ROS) that play an important role in muscle function. Moreover, dysfunctional mitochondria trigger catabolic signaling pathways which feed-forward to the nucleus to promote the activation of muscle atrophy. Exercise, on the other hand, improves mitochondrial function by activating mitochondrial biogenesis and mitophagy, possibly playing an important part in the beneficial effects of physical activity in several diseases. Optimized mitochondrial function is strictly maintained by the coordinated activation of different mitochondrial quality control pathways. In this review we outline the current knowledge linking mitochondria-dependent signaling pathways to muscle homeostasis in aging and disease and the resulting implications for the development of novel therapeutic approaches to prevent muscle loss.

## Introduction

Nearly 40–50% of total body mass in non-obese mammals is composed by skeletal muscles. Striated muscles are plastic tissues that can undergo adaptive changes in their structure or functional properties to meet new challenges. For example, muscle mass can either increase or decrease in response to metabolic demands like exercise or inactivity. Moreover, muscle strength or resistance to fatigue can be modulated by training or detraining. The regulation of muscle size, due to its limited proliferative capacity, is determined by the coordinated balance between protein synthesis and protein degradation. Mechanical overload or anabolic hormonal stimulation shifts the balance toward protein synthesis with consequent increases in fiber size, a process called hypertrophy. Conversely, in catabolic conditions protein degradation exceeds protein synthesis leading to muscle weakness and muscle atrophy. The decrease in cell size is mainly due to loss of organelles, cytoplasm and proteins. Skeletal muscle atrophy occurs in several pathological conditions like disuse, denervation, immobilization, sepsis, burn injury, cancer, AIDS, diabetes, heart and renal failure and during aging. Importantly, the decrease of muscle mass increases morbity, impairs the efficacy of many therapeutic treatments and contributes to mortality. Muscle mass is mainly controlled by two major signaling pathways: TGFβ/Smads and IGF1-AKT-mTOR-FoxO. TGFβ superfamily of ligands regulate the size of muscles via the Smad transcription factors (Sandri, 2013). Smad2/3 control catabolic genes while Smad1/5/8 regulate anabolic genes (Sartori et al., 2013, 2014). The IGF1-AKT- mTOR axis increases protein synthesis by stimulating the translational machinery while simultaneously blocking FoxOs transcription factors and protein degradation pathways (Sandri, 2013). Two main ATP-dependent proteolytic systems are activated during muscle atrophy in order to contribute to muscle loss. The ubiquitin-proteasome system degrades predominantly myofibrillar proteins, whereas the autophagy-lysosome system removes dysfunctional organelles, protein aggregates as well as unfolded and toxic proteins. Muscle atrophy requires the activation of gene transcription programs that regulate the expression of a subset of genes that are named atrophy-related genes or atrogenes (Bodine et al., 2001; Gomes et al., 2001; Lecker et al., 2004; Sandri et al., 2004; Sacheck et al., 2007). These atrogenes belong to several fundamental biological processes such as the ubiquitin-proteasome and autophagy-lysosome systems, protein syntesis, ROS detoxification, DNA repair, unfolding protein response (UPR), mitochondria function and energy balance. FoxO family of transcription factors (FoxO1, FoxO3, and FoxO4), which are targets of AKT, are key mediators of the catabolic response during atrophy (Sandri et al., 2004; Mammucari et al., 2007; Milan et al., 2015). Indeed, specific inhibition of FoxOs in muscle protects from cancer cachexia-, fasting- or denervation-induced atrophy (Judge et al., 2014; Milan et al., 2015) as they are critical for the regulation of several atrogenes. Moreover, at least half of the atrogenes require FoxOs for their up or downregulation (Milan et al., 2015). FoxO-dependent atrogenes include the E3 Ubiquitin ligases Atrogin-1, MuRF-1, MUSA1, SMART, and several autophagy-related genes such as LC3, GABARAPL1, BNIP3, CATHEPSIN L (Bodine et al., 2001; Sandri et al., 2004; Mammucari et al., 2007; Milan et al., 2015). Therefore, FoxOs coordinate both major proteolytic systems of the cell, the autophagy-lysosome and the ubiquitin-proteasome.

Interestingly, in addition to genes linked with proteolytic pathways, more than 10% of the atrophy-related genes are directly involved in energy production. Furthermore, several genes coding for enzymes important in glycolysis and oxidative phosphorylation are coordinately suppressed in atrophying muscles (Lecker et al., 2004). These interesting findings suggest that alterations in mitochondria and the mitochondrial network morphology can have potential deleterious consequences for the maintenance of muscle mass and function. Recent data shows that the mitochondrial network communicates with myonuclei to adapt muscle function to the physiological or pathological demands. Here we will review the principal pathways that control mitochondrial quality and the importance of mitochondrial network in the regulation of muscle mass and metabolism.

## Optimization of mitochondrial function: a quality control issue

**Figure 1:**
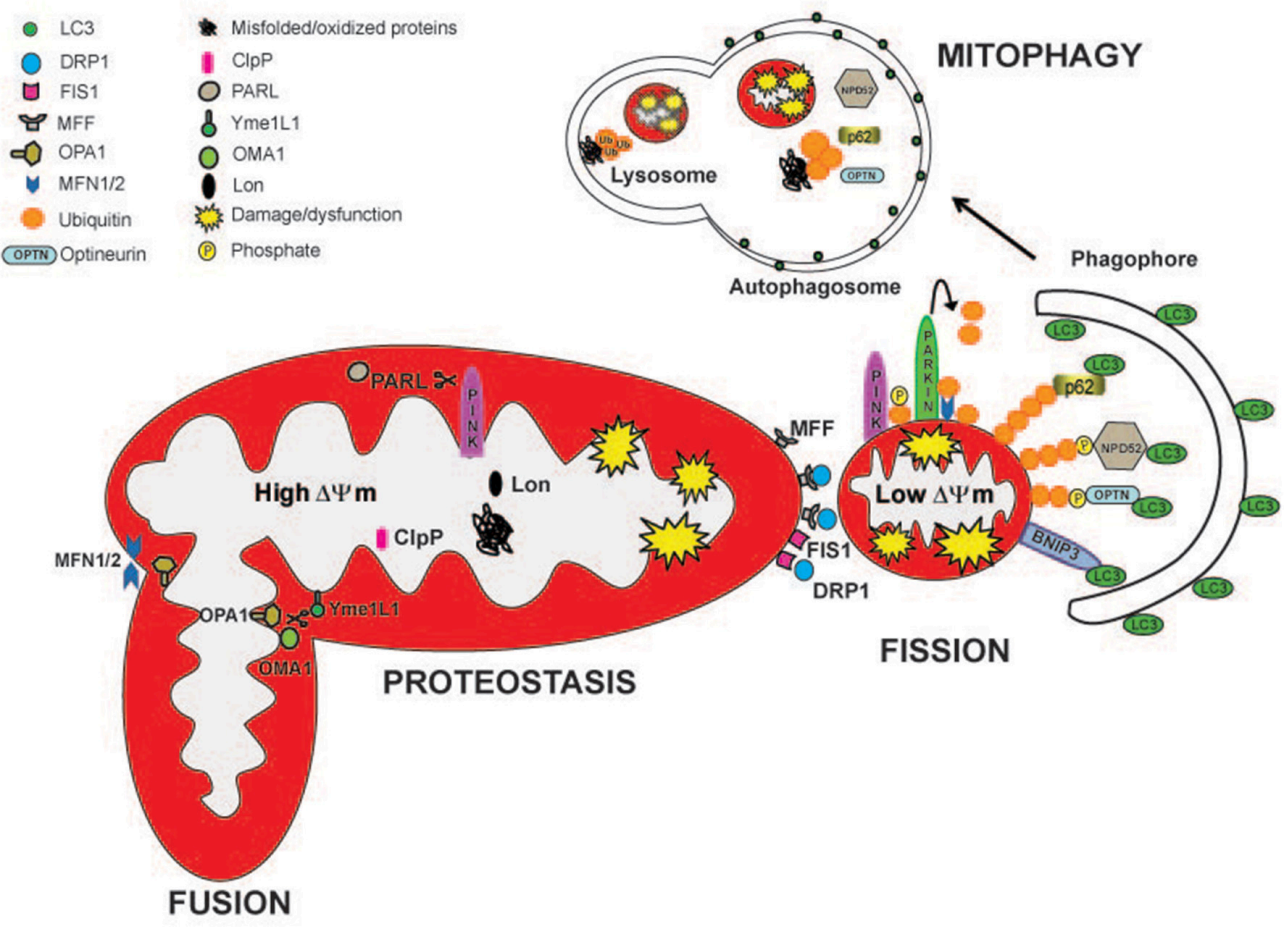
**Mitochondrial quality control pathways are depicted**. The actions of specific mitoproteases, Lon, ClpP, Oma1, Yme1L1, and PARL, maintain mitochondrial proteostasis and regulate mitochondrial function. PARL, Oma1, and Yme1L1 control mitochondrial dynamics by proteollytically processing Opa1 protein, which is important for mitochondrial fusion and cristae remodeling. PARL degrades PINK1, regulating mitophagy. Drp1, Fis, and Mff are the major proteins involved in mitochondrial fission. Partially damaged mitochondrial network divides into two fragments with different ΔΨm. The fragment with higher ΔΨm rejoins the functional mitochondrial network through mitochondrial fusion. Depolarized organelles will be removed by mitophagy. Bnip3 acts as a mitophagic receptor, binding to LC3 to tether mitochondria to the autophagosome. PINK1 accumulates on the surface of depolarized mitochondria where it phosphorylates ubiquitinated OMM proteins and the Parkin UBL domain. Parkin will further promote the ubiquitination of the outer mitochondrial membrane proteins, like MFN1/2. Then, the ubiquitinated proteins can be recognized by the adaptors p62/SQSTM1, Optineurin (OPTN), and NPD52 to initiate mitophagy.

Mitochondria are continuously challenged by reactive oxygen species (ROS), an inexorable by-product of oxidative phosphorylation. In order to prevent an excessive production of ROS but also the release of dangerous factors such as cytochrome c, AIF or endonuclease G, mammalian cells contain several systems that maintain mitochondrial integrity and function. This mitochondrial quality control includes pathways related to protein folding and degradation as well as systems involved in organelle shape, movement and turnover (Figure 1). The activation of each specific quality control system depends on the degree of mitochondrial damage.

### Mitochondrial proteolysis controls both mitochondrial protein turnover and mitochondrial function

Proteomic studies identified in human mitochondria approximately 1200 proteins (Calvo et al., 2015). However, only 1% of these proteins is encoded by mitochondrial DNA (mtDNA) (13 proteins in humans). Therefore, the remaining 99% is encoded by nuclear DNA (nDNA) genes, which are synthesized by ribosomes in cytoplasm and are imported into mitochondria (Harbauer et al., 2014). The incorporation of these precursors into specific mitochondria subcompartments such as the outer mitochondrial membrane (OMM), the intermembrane space (IMS), the inner mitochondrial membrane (IMM) and the mitochondrial matrix requires a specific and highly regulated import machineries (Harbauer et al., 2014). To maintain the proteostasis in each mitochondrial compartment, specific proteases (mitoproteases) will rapidly degrade misfolded or oxidized proteins. Mitoproteases-mediated quality control is the first line of defense against a mild mitochondrial damage. In the mitochondrial matrix, protein turnover is controlled by 3 AAA proteases: the soluble Lon and ClpP and the membrane-bound m-AAA. Protein degradation in the IMS is controlled by the membrane-bound i-AAA Yme1L1, the soluble HtrA2/Omi, the metallopeptidases OMA1 and the rhomboid protease PARL. Different reports showed that these mitoproteases do not only monitor mitochondrial protein quality but they can also decide mitochondrial fate. For example, m -AAA, Yme1L1, HtrA2, OMA1, and PARL l cleave the profusion protein Opa1 affecting mitochondrial morphology and function (Alavi and Fuhrmann, 2013). In addition, PARL modulates mitophagy by degrading the mitophagy protein PINK1 (Jin et al., 2010). For a review on the roles and the pathological relevance of these mitoproteases please refer to Quiros et al. (2015). Beside subcompartment-specific proteases, mitochondrial protein turnover is regulated also by the cytosolic ubiquitin-proteasome system (UPS). The UPS marks proteins for proteasomal degradation by the covalent linkage of a chain of ubiquitin proteins. A proteomic study in mouse cardiac muscle identified numerous OMM as well as IMS, IMM, and matrix proteins to be ubiquitinated (Jeon et al., 2007). A still unanswered question is how the cytosolic proteasome can degrade integral mitochondrial membrane proteins. During ER stress, misfolded proteins accumulate into the ER lumen and need to be retrotranslocated into the cytosol where they are flagged with ubiquitin and degraded by the proteasome in a process called ER-associated protein degradation (ERAD). Mitochondria have an ERAD-like mechanism, the mitochondria-associated degradation (MAD) pathway (Heo et al., 2010). ERAD and MAD pathways share some key components like the AAA ATPase p97 and the cofactor Npl4 (Ye et al., 2003; Heo et al., 2010). p97/Npl4 complex regulates the retrotranslocation of proteins from both the ER (Ye et al., 2001, 2003) and OMM (Heo et al., 2010; Tanaka et al., 2010; Xu et al., 2011). In mitochondria, p97 provides the driving force to extract Mfn1, Mfn2 and the anti-apoptotic protein Mcl1 from the OMM and chaperoned them for their degradation by the proteasome in the cytosol (Tanaka et al., 2010; Xu et al., 2011). Several components of the UPS are recruited to mitochondria such as the E3 ligases Parkin, March V/Mitol, Huwe, and Mulan (Livnat-Levanon and Glickman, 2011). These mitochondrial E3 ligases are a good example of the synergistic crosstalk between different mitochondria quality control systems. For instance, the activity of these ubiquitin ligases impacts on mitochondrial morphology and half-life. Indeed, Parkin-mediated ubiquitination of Mfn1, Mfn2, and VDAC1 blocks/reduces mitochondrial fusion and promotes mitophagy (Narendra et al., 2008; Gegg et al., 2010; Geisler et al., 2010; Tanaka et al., 2010). On the other hand, in physiological conditions Parkin avoid mitochondrial fragmentation by repressing Drp1 levels (Wang et al., 2011). Finally, MarchV/Mitol ubiquitinates Drp1, Fis1, and mitofusins 1 and 2 (Nakamura et al., 2006; Yonashiro et al., 2006; Karbowski et al., 2007). One intriguing issue is whether intramitochondrial proteins that do not face the cytosol can be substrates of the MAD pathway. Even though a retrotranslocation machinery has not been identified, some evidence indicates that the UPS controls IMM proteins. In fact, as a consequence of proximity to the respiratory chain, IMM proteins are exposed to ROS generated by mitochondrial respiration and therefore, oxidized. For example, the mitochondrial uncoupling protein 2 (UCP2) (Azzu and Brand, 2010; Bragoszewski et al., 2013) and the subunit of the OXPHOS complex V OSCP have been found to be retrotranslocated to the OMM for ubiquitination and proteasome degradation (Margineantu et al., 2007). Indeed, proteasome inhibition leads to accumulation of the IMM proteins UCP2, COXI, III, IV, OSCP (Margineantu et al., 2007; Azzu and Brand, 2010). Although the role of the UPS in different cellular processes has been well characterized, studies concerning the interaction with mitochondria are only at the beginning. Several critical issues related to how substrates are identified and how they are transported out for degradation need to be solved in the future.

Under stress conditions, when the degradation pathways are not sufficient to blunt the damage and restore a normal mitochondrial function, a retrograde signal is activated which coordinates nuclear gene expression. This mitochondria-to-nucleus response is named mitochondrial unfolding protein response (UPR^mt^) (Zhao et al., 2002). The ultimate purpose of UPR^mt^ is to maintain proteostasis by promoting the expression of chaperones to improve protein folding, inhibit protein synthesis to alleviate ER stress and to induce expression of m-AAA proteases like ClpP and Yme1 to remove damaged proteins. For reviews on this topic see (Jovaisaite and Auwerx, 2015; Schulz and Haynes, 2015).

### Mitochondrial dynamics: not only a matter of shape

Mitochondrial dynamics is defined by the capacity of the organelle to rapidly change its size, shape and distribution by continuously alternating fusion and fission events. Fusion leads to elongated mitochondria with increased interconnectivity into a tubular network. On the contrary, fission fragments the network into unconnected shorter organelles. In physiological conditions, most mammalian cells show a continuous filamentous mitochondrial network with the exception of cardiomyocytes that display fragmented mitochondria that do not form a network (Song and Dorn, 2015). However, the mitochondrial pool rapidly changes its morphology according to the cellular needs. Increased mitochondrial fusion facilitates the redistribution of metabolites, proteins and mtDNA within the network. Moreover, fusion between healthy and damaged organelles allows to dilute the damaged material into the healthy network, avoids the accumulation of dysfunctional mitochondria and maintains their overall fitness (function) (Twig et al., 2008). On the other hand, mitochondrial fragmentation is a mechanism that segregates dysfunctional or damaged components of the mitochondrial network, allowing their removal via mitophagy (Otera and Mihara, 2011).

However, excessive fission generates isolated mitochondria which are less efficient in ATP production and are dysfunctional because they consume ATP to maintain their membrane potential (Benard et al., 2007).

#### Fusion machinery

Mitochondrial structure is optimized to ensure respiration and ATP production with a minimal release of ROS. This architecture must be preserved during fusion and fission events. Therefore, when mitochondria fuse they follow a scheme which starts with mitochondrial tethering, continues with fusion of OMM and concludes with fusion of IMM. In mammals, the membrane-bound GTPases mitofusin 1 (Mfn1), mitofusin 2 (Mfn2), and optic atrophy protein 1 (Opa1) are required for fusion of the OMM and IMM respectively.

##### OMM profusion proteins

Mfn1 and Mfn2 are integrated into the OMM and have two cytosolic heptad repeat domains (HR1 and HR2) which interact to form a dimeric anti parallel coiled-coil structure. Both mitofusins can consequently interact homotypically (Mfn1-Mfn1, Mfn2-Mfn2) or heterotypically (Mfn1-Mfn2) to promote the tethering of different adjacent mitochondria and fusion of the OMMs (Koshiba et al., 2004). These proteins have a high degree of homology but they do not have the same functions. Mfn1 has higher GTPase and organelle fusion activity while Mfn2 has greater affinity for GTP (Ishihara et al., 2004). Aditionally, Mfn2 and not Mfn1 is expressed on the mitochondria-associated endoplasmic reticulum membranes (MAM) and to a lesser extent to the endoplasmic/sarcoplasmic reticulum (ER/SR) (de Brito and Scorrano, 2009). This unique distribution allows for the close communication between the two organelles. Indeed, Mfn2 bridges mitochondria to ER/SR facilitating important processes linked to ER-mitochondria interactions like calcium homeostasis and signaling (de Brito and Scorrano, 2009; Ngoh et al., 2012; Sebastian et al., 2012; Munoz et al., 2013; Ainbinder et al., 2015). Recent data indicates Mfn2 as a modulator of the UPR during ER stress. In fact, Mfn2 deficiency leads to fragmented ER network and ER stress (Ngoh et al., 2012; Sebastian et al., 2012; Munoz et al., 2013; Bhandari et al., 2015) by chronic activation of PERK (Munoz et al., 2013). Mfn2, like the other mitochondria-shaping proteins is not free from post-translational modifications. For instance, when Mfn2 is phosphorylated by PINK1, it becomes a receptor for the ubiquitin ligase Parkin (Chen and Dorn, 2013). In addition, the E3 ligases HUWE1 and Mul1 also promote Mfn2 ubiquitination and proteasomal degradation (Leboucher et al., 2012; Lokireddy et al., 2012). Conversely, the formation of a Lys63- polyubiquitin chain by the E3 ligase MARCHV/Mitol does not induce Mfn2 degradation but increases its activity as well as MAM formation and function (Sugiura et al., 2013). Altogether, Mfn2 promotes mitochondrial fusion, enhances ER-mitochondria and activates mitophagy via PINK1/Parkin pathway.

##### Opa1: the pleiotropic IMM fusion protein

The profusion protein Opa1 is regulated by proteolytic processing. There are several splicing variants of Opa1 (8 in humans and 4 in mice) which are expressed in a tissue specific manner. Some of them are cleaved to generate soluble short Opa1 (Opa1S) from long Opa1 isoforms (Opa1L) (Ishihara et al., 2006). Opa1L is anchored to the IMM by a transmembrane domain at the N-terminus and can be further cleaved in exon 5 (site S1) by OMA1, a process that is mitochondrial membrane potential (ΔΨm)-dependent (Ehses et al., 2009; Head et al., 2009). The mitoprotease Yme1 cut the site S2 in exon 5b of a subset of Opa1L belonging to the splicing variants 4, 6, 7, and 8 (Anand et al., 2014). As aforementioned, other mitoproteases such as m -AAA, HtrA2, and PARL l are also involved in Opa1 processing (Alavi and Fuhrmann, 2013). Furthermore, Opa1 is cleaved in its C-terminal region by an unknown cysteine protease in the post-prandial liver, in a process dependent on Mfn2 and independent of OMA1 (Sood et al., 2014). Opa1-dependent mitochondrial fusion needs Mfn1 (Cipolat et al., 2004) as well as Opa1S and Opa1L forms (Frezza et al., 2006; Song et al., 2007). However, under stress conditions fusion can rely only on Opa1L while Opa1S forms are dispensable (Tondera et al., 2009; Anand et al., 2014). In addition, oligomerization of soluble and membrane-bound OPA1 isoforms controls cristae remodeling and the assembly of respiratory chain complexes into supercomplexes, a structure that enhances mitochondrial respiration (Cogliati et al., 2013). On the contrary, complete conversion of Opa1L into Opa1S inhibits fusion and increases mitochondrial fission (Ishihara et al., 2006; Anand et al., 2014).

#### Fission machinery

Mitochondrial fission depends on the cytosolic GTPase dynamin-related protein 1 (Drp1). Drp1 is recruited to the OMM where it assembles into multimeric ring complexes that form active GTP-dependent mitochondrial fission sites (Smirnova et al., 2001). Drp1 lacks hydrophobic membrane-binding domains and for this reason its recruitment on OMM is dependent on mitochondrial membrane proteins that act as receptors. Accordingly, Drp1 interacts with the integral OMM proteins: Fis1, Mff, mitochondrial elongation factor 2/mitochondrial dynamics protein 49 (MIEF2/MiD49) and MIEF1/MiD51. Fis1 is the major Drp1 receptor in yeasts (Karren et al., 2005). However, increasing evidence suggests that in mammals Fis1 is not the only OMM-anchor receptor of Drp1. For example, downregulation of Fis1 leads to mitochondrial elongation, even if the recruitment of Drp1 to mitochondria is not reduced (Lee et al., 2004). Furthermore, conditional deletion of Fis1 in a carcinoma model did not lead to defects in mitochondrial fission, indicating that Fis1 is not essential for fission (Otera et al., 2010). Recently, new components of the mammalian fission machinery were identified (Otera et al., 2010; Palmer et al., 2011; Zhao et al., 2011). Mff, the OMM-anchored mitochondrial fission factor, colocalizes with Drp1 on mammalian mitochondria. Its overexpression promotes mitochondrial fission with increased Drp1 recruitment to mitochondria. On the contrary, silencing Mff generates elongated mitochondria and Drp1 cytosolic distribution. Moreover, Drp1 affinity for Mff is higher than for Fis, suggesting that Mff preferentially functions as Drp1 receptor (Otera et al., 2010). MIEF1/MiD51 and the variant MIEF2/MiD49 recruit Drp1 to mitochondria independently of Fis1or Mff. Moreover, overexpression of MiD51 does not induce mitochondrial fragmentation but instead prevents mitochondrial fission by sequestering and inhibiting Drp1 (Palmer et al., 2011, 2013; Zhao et al., 2011; Loson et al., 2013). MIEF1/MiD51 preferentially binds Fis1 but can also interact with DRP1 and consequently, depending from the levels of Fis1, two different protein complexes are generated. When the level of Fis1 is high, it sequesters MIEF1/MiD51 that cannot anymore interact and inhibit DRP1 allowing mitochondrial fission. In contrast, the downregulation of Fis1 releases MIEF1/MiD51 that can now block Drp1 resulting in mitochondrial fusion (Zhao et al., 2011). Finally, in yeast Drp1 has been suggested to act as a regulatory factor not only of fission but also of fusion (Huang et al., 2011). In fact, it has been reported that mitochondria-localized Drp1 can interact with the HR1 domain of MFN2 to promote mitochondrial fusion (Huang et al., 2011).

##### DRP1 regulation

The complexity of Drp1 regulation has been recently reviewed (Elgass et al., 2013; Otera et al., 2013), here we will briefly summarize some critical steps. Drp1-dependent mitochondrial fission is regulated by post-translational modifications like ubiquitination, phosphorylation and SUMOylation to ensure a rapid adaptation to cellular needs (Otera et al., 2013). For example, Drp1 is targeted for proteasomal degradation by Parkin-mediated ubiquitination (Wang et al., 2011) while the ubiquitination mediated by MARCHV/Mitol is controversial with different outcomes depending on cell context (Nakamura et al., 2006; Karbowski et al., 2007). Sumoylation of Drp1 by the SUMO E3 ligase mitochondria-anchored protein ligase (MAPL) stimulates mitochondrial fission (Braschi et al., 2009). Phosphorylation of different residues of DRP1 cause opposing effects. During mitosis, when organelles are inherited by daughter cells, mitochondrial fission activity is promoted by Cdk1-cyclin B-dependent Drp1 phosphorylation at Ser616 (in humans) in the GTPase effector domain (GED) (Taguchi et al., 2007). However, Drp1 activity is also induced by the posphorylation of Ser637 in the GED by the calcium/calmodulin-dependent protein kinase I alpha (CaMK1 alpha). As a result, this phosphorylation increases mitochondrial translocation of Drp1 because it facilitates the interaction with Fis1 (Han et al., 2008). However, phosphorylation of the same Ser637 by the protein kinase A (PKA) has an opposite effect causing DRP1 retention in the cytosol (Cribbs and Strack, 2007). This inhibition is counteracted by the calcium dependent phosphatase, calcineurin (Cribbs and Strack, 2007; Cereghetti et al., 2008). An interesting model of how post-translational modulation of fission can impinge on both, fusion and autophagy is what happens during early stages of starvation. Under this condition, mitochondrial fission is impaired due to the inhibition of Drp1. Two different reports indicate that starvation in cells leads to increased phosphorylation of Ser637 due to PKA activity as well as to decreased phosphorylation of Ser616. As a consequence, Drp1 is retained in the cytosol and mitochondria are elongated and spared from autophagic degradation. The resulting tubular mitochondrial network displays an increased number and density of cristae and presents more dimers of the ATP synthase (Gomes et al., 2011; Rambold et al., 2011). In conclusion, the shaping machinery adapts mitochondrial morphology to the bioenergetic requirements of the cell determining the homeostasis and the fate of the cell. Even though, our knowledge of the mechanisms controlling mitochondria dynamics is growing, many aspects of this process are still unclear.

### Lysosomal-dependent degradation of mitochondria: mitochondria-derived vescicles (MDV) vs. mitophagy

Depending from the type of injury, mitochondria can adopt two different routes for delivering the damaged components to lysosome for degradation. When mitochondria deterioration is mild without global mitochondrial depolarization, local removal of mitochondrial content can be done by the generation of vesicles that bud-off from mitochondria and contain matrix components (Soubannier et al., 2012a,b). These mitochondria-derived vescicles (MDV) are 70–150 nm in size and do not require the fission machinery for their biogenesis. MDVs carry oxidized proteins coming from different mitochondrial compartments and are directed to lysosomes (Soubannier et al., 2012b). Therefore, mitochondrial oxidative stress induces MDVs via Pink-Parkin system (McLelland et al., 2014) independently of the autophagy-related proteins Atg5, Rab9, or Beclin (McLelland et al., 2014). Since Drp1 fission activity is not required for MDV budding (Soubannier et al., 2012a,b), the identification of the machinery necessary for MDV biogenesis is still unknown. This system works in conjunction with other mitochondria quality control pathways like mitochondrial proteolysis and dynamics and precedes mitophagy. In fact, when mitochondria are irreversibly damaged, they are excised from the mitochondrial network by the fission machinery and sequestered into autophagic vesicles for their degradation in the lysosome. This selective mitochondrial degradation via autophagy is called mitophagy. Apart from removal of dysfunctional mitochondria, mitophagy is essential for mitochondria turnover in the basal state and during the development of specialized cells such as red blood cells (Sandoval et al., 2008). The loss of mitochondrial membrane potential is a major trigger for mitophagy (Elmore et al., 2001). The selectivity of mitophagy is controlled by mitochondria dynamics and by the proteins PINK1, Parkin, Bnip3L/Nix, and Bnip3.

Recessively inherited forms of Parkinson's disease are associated with loss-of-function mutations of the PTEN-induced kinase 1 (PINK1) and the E3 ubiquitin ligase Parkin. Under basal conditions Pink1 is imported into the IMM where it is cleaved by PARL in a voltage dependent manner. The resulting fragments will be further degraded by the cytosolic UPS (Jin et al., 2010). Therefore, healthy mitochondria have undetectable levels of PINK1. However, when ΔΨm is lost, full length PINK1 is not further imported and accumulates on OMM. Here, PINK1 phosphorylates ubiquitin at Ser65 of ubiquitinated OMM proteins and the ubiquitin-like domain of Parkin (Kane et al., 2014; Kazlauskaite et al., 2014; Koyano et al., 2014). Once phosphorylated, Parkin enhances the mitophagy signal by generating more ubiquitin chains on OMM proteins that can be further substrates for PINK1 (Lazarou et al., 2015). Subsequently, the autophagic adaptors optineurin, NDP52 (Lazarou et al., 2015) bind the phosphorylated ubiquitins tagging the OMM proteins with LC3 to initiate mitophagy. The contribution of the autophagy adaptor p62/SQSTM1 has been found to be dispensable for mitophagy but important for perinuclear clustering of depolarized mitochondria (Narendra et al., 2010; Lazarou et al., 2015). However, these data were obtained *in vitro* and thus the physiological relevance *in vivo* needs to be validated. For instance, while p62 and NBR1 are well expressed in adult muscles, optineurin and NDP52 proteins are barely detectable (Nicot et al., 2014; Lazarou et al., 2015). Bnip3 and Nix (also called Bnip3L) belong to the BH3-only proteins and are implicated in both apoptosis and mitophagy. These proteins translocate to mitochondria, form homodimers and disrupt mitochondrial membrane potential (Sandoval et al., 2008). They contain two evolutionary conserved LIR (LC3 Interacting Region) domains to bind LC3 and GABARAP. Nix preferentially interacts with GABARAP whereas Bnip3 binds LC3 (Novak et al., 2010; Hanna et al., 2012). These results indicate that Bnip3 and Nix have an important role in mitochondrial turnover, acting as autophagy receptors which tether the mitochondria to the autophagosome. Moreover, the induction of autophagy by the overexpression of Bnip3 in cardiomyocytes requires Drp1 translocation to mitochondria to mediate mitochondrial fission (Lee et al., 2011).

## Unraveling the mechanisms linking mitochondrial fitness to muscle health

Energy requirements during contraction in skeletal muscles increase instantaneously up to 100-fold the consumption of ATP (Blei et al., 1993; Gaitanos et al., 1993). Therefore, to support this high demand of ATP, skeletal and cardiac muscles rely on oxidative phosphorylation for energy production. Indeed these tissues contain the greatest amount of mitochondria. Several reports identified within the myofibers two distinct mitochondrial populations that differ in their subcellular localization, morphology as well as biochemical and functional properties (Howald et al., 1985; Hoppeler et al., 1987; Romanello and Sandri, 2013). Subsarcolemmal (SS) mitochondria are located just underneath the sarcolemma and have a large, lamellar shape. In contrast, the intermyofibrillar (IMF) mitochondria are smaller, more compact, and located between the contractile filaments. However, the existence of two separated mitochondrial pools was challenged by recent findings on high-resolution, three-dimensional electron microscopy (FIB-SEM). Dahl and collaborators showed that SS and IMF are physically connected in human muscles (Dahl et al., 2015). Moreover, Ballaban's lab identified 4 different mitochondrial morphologies: the paravascular mitochondria (PVM), I-band mitochondria (IBM), fiber parallel mitochondria (FPM), and cross fiber connection mitochondria (CFCM) (Glancy et al., 2015). All these mitochondria are highly interconnected forming a conductive pathway that can rapidly distribute energy in the form of electrical conduction throughout myofibers, enabling muscles to respond immediately to changes in energy requirements.

### Exercise and peroxisome proliferator-activated receptor-γ coactivator-1 (PGC-1) family members

There is compelling evidence that regular exercise has many beneficial effects for human health and life span (Neufer et al., 2015). The effects of exercise are sensed by most organs including cardiovascular, neuroendocrine, respiratory and musculoskeletal systems. Importantly, some forms of exercise training counteract skeletal muscle loss (Hourde et al., 2013; Cohen et al., 2015). On the other hand, physical inactivity has been identified as the fourth risk factor of morbidity/disability and mortality (Neufer et al., 2015). Beneficial adaptations to endurance exercise training (moderate intensity with prolonged duration) in skeletal muscle include the activation of transcriptional dependent pathways that impinge on mitochondrial biogenesis (Little et al., 2011), mitochondrial quality and mitophagy (Grumati et al., 2011; He et al., 2012a; Lo Verso et al., 2014).

#### PGC-1α and PGC-1β

Mitochondrial biogenesis requires a coordinated action on nuclear and mitochondrial genomes that is regulated by the coactivators PGC-1α and PGC-1β (peroxisome proliferator-activated receptor-γ coactivator-1α and β) PGC-1 family members are preferentially expressed in tissues enriched in mitochondria like heart, adipose tissue and slow-twitch skeletal muscle. Exercise, fasting and cold exposure highly regulate their expression. Since PGC-1α and PGC-1β are coactivators that lack DNA binding domains, they elicit their function by modulating the activity of several transcription factors including PPARs, nuclear respiratory factors (NRFs), myocyte enhancing factors (MEFs), estrogen-related receptor (ERR), forkhead box (FoxOs), and yin-yang (YY1) (Lin et al., 2005; Olesen et al., 2010; Zechner et al., 2010). Exercise greatly stimulates PGC-1α via a transcription-dependent upregulation and by several post-translational modifications like phosphorylation and deacetylation (Wright et al., 2007; Cantó et al., 2009; Egan et al., 2010). Moreover, acute endurance exercise promotes the translocation of PGC-1α from the cytosol to the nucleus (Wright et al., 2007) and mitochondria (Safdar et al., 2011). This subcellular relocalization helps nuclear and mitochondrial crosstalk to promote mitochondrial biogenesis (Safdar et al., 2011). Indeed, both Tfam and nuclear gene products are imported into mitochondria where they regulate the expression of mitochondrial proteins required for ATP synthesis. Interestingly, deletion of PGC-1α or PGC-1β in mice showed mild phenotypes (Leone et al., 2005; Lelliott et al., 2006; Vianna et al., 2006; Sonoda et al., 2007). Although, exercise capacity was found reduced in PGC-1α knockout mice (Leone et al., 2005; Handschin et al., 2007; Wende et al., 2007), exercise-induced mitochondrial adaptations in PGC-1α knockout mice were similar to wild-type animals (Leick et al., 2008; Geng et al., 2010). These confilicting results can be probably explained because these two coactivators are redundant and partially compensate each other. In fact, when PGC-1β is deleted on a generalized PGC-1α-deficient background (Zechner et al., 2010) it was shown that these factors share a subset of target genes and display overlapping functions. Accordingly, exercise performance, in this model, is greatly decreased compared to single PGC-1α or PGC-1β knockout animals (Zechner et al., 2010). PGC-1α and PGC-1β are necessary for the maintenance of mitochondrial function (Zechner et al., 2010). Therefore, the deletion of both, PGC-1α and PGC-1β induce severe mitochondrial dysfunction that leads to rapid depletion of glycogen stores and to premature fatigue during exercise (Zechner et al., 2010). Moreover, PGC-1α and PGC-1β control mitochondrial dynamics by stimulating Mfn1 and Mfn2 gene expression in a ERRα-dependent manner (Soriano et al., 2006; Russell et al., 2013). Accordingly, Mfn1, Mfn2, and Drp1 are severely downregulated in PGC-1α and PGC-1β muscle-specific knockout mice (Zechner et al., 2010). Moreover, PGC-1α fine tunes autophagy regulation during some forms of disuse atrophy (Vainshtein et al., 2015). Therefore, PGC-1 coactivators not only increase mitochondrial content but also mitochondrial quality by modulating fusion/fission processes and mitophagy. Surprisingly, PGC-1α and PGC-1β muscle-specific knockout mice do not exhibit defects in muscle fiber type formation in terms of myosin content (Zechner et al., 2010). This is in contrast with a previous study that showed an increase of type I fibers in transgenic mice that specifically express PGC-1α in fast glycolytic muscles (Lin et al., 2002). PGC-1α mRNA levels drop in different atrophying conditions like denervation (Sandri et al., 2006), hind limb suspension (Cannavino et al., 2015) aging (Chabi et al., 2008) or type 2 diabetic patients (Patti et al., 2003). Importantly, maintaining PGC-1α levels high during catabolic conditions, either by the use of transgenic mice or by transfecting adult muscle fibers, spares muscle mass during hind limb suspension, sarcopenia, cardiac cachexia, denervation, fasting, or expression of constitutively active FoxO3 (Sandri et al., 2006; Wenz et al., 2009; Geng et al., 2011; Cannavino et al., 2015). Similar beneficial effects have been recently obtained by overexpression of PGC-1β, an homolog of PGC-1α (Brault et al., 2010). The positive action on muscle mass of these cofactors is caused by the inhibition of the autophagic-lysosomal and the ubiquitin proteasome degradation pathways. PGC-1α and β reduce protein breakdown by inhibiting the transcriptional activity of FoxO3 and NF-κB, but they do not affect protein synthesis (Brault et al., 2010). Thus, these cofactors can prevent the excessive activation of proteolytic systems by inhibiting the action of the pro-atrophy transcription factors without perturbing the translational machinery.

#### PGC-1α4

A new splicing variant transcript from PGC-1α gene, PGC-1α4, was identified and shown to be involved in the regulation of muscle mass (Ruas et al., 2012). PGC-1α4 expression is induced with resistance exercise protocols (fewer repetitions at high muscle loads aimed at increasing muscle mass) and not with endurance exercise. Skeletal muscle-specific transgenic overexpression of PGC-1α4 results into hypertrophic and stronger mice (Ruas et al., 2012). PGC-1α4 controls muscle mass by inducing IGF1 and repressing the myostatin pathway. Importantly, PGC-1α4 overexpression counteract muscle loss induced by hindlimb suspension and cancer-cachexia (Ruas et al., 2012). However, the results obtained by Ruas and collegues are in contrast with different reports analyzing exercise-mediated adaptations in human muscles (Ydfors et al., 2013; Lundberg et al., 2014; Silvennoinen et al., 2015). In humans, PGC-1α4 is regulated transiently with exercise regardless of mode (resistance or endurance) (Ydfors et al., 2013; Lundberg et al., 2014; Silvennoinen et al., 2015). Moreover, chronic training adaptations, such as increases in muscle mass and force do not correlate with changes in the expression of PGC-1α4 after resistance exercise or after the combination of aerobic and resistance exercise (Lundberg et al., 2014).

### Calcium and AMPK signaling pathways

The beneficial effects of exercise can be also mediated by contraction-induced changes in calcium homeostasis, ATP consumption and ROS production. The alteration in calcium levels activates calcium-sensitive signaling such as calcium/calmodulin-dependent protein kinases (CaMK) and calcineurin/NFAT systems that are important for fiber type regulation and muscle plasticity (Wu et al., 2002; Blaauw et al., 2013).

ER-mitochondria crosstalk is essential for excitation-calcium coupling during muscle contraction. Indeed a great amount of ATP produced by mitochondria is consumed by SERCA pumps. Moreover, the positioning of intermyofibrillar mitochondria nearby to SR makes them affected by the calcium wave of contraction. In this context, the role of mitochondrial calcium uptake in the maintenance of skeletal muscle mass was recently investigated (Mammucari et al., 2015). *In vivo* gain- and loss-of-function experiments in skeletal muscle showed that modulation of mitochondrial calcium uniporter (MCU) controls mitochondrial volume. Overexpression of MCU in muscles leads to increased protein synthesis with a resulting hypertrophic phenotype which impinge on IGF1/AKT and PGC-1α4 pathways. Moreover, MCU overexpression protects from denervation-induced atrophy. Supporting the importance of MCU in skeletal muscle is the identification of mutations of MICU1, an MCU regulator, in patients with proximal muscle weakness, learning difficulties and extrapiramidal motor disorder (Logan et al., 2014). In addition, different reports linked calcium homeostasis with the control of muscle mass (Blaauw et al., 2013). Therefore, the investigation of calcium entry pathways in skeletal muscle mitochondria open the possibility to better understand the role of calcium in ER-mitochondria communication and its impacts on muscle mass regulation.

On the other side ATP depletion modifies the AMP/ATP ratio, activating the energy sensor AMP-activated protein kinase (AMPK). The activation of this signal transducer is crucial for the metabolic adaptations in response to energy stress. AMPK inhibition of anabolic pathways together with stimulation of catabolic pathways are aimed at conserving/restoring ATP (Ha et al., 2015). In skeletal muscle AMPK activation is sufficient to increase glucose uptake (by increasing GLUT4 translocation), fatty acid oxidation and mitochondrial biogenesis. In fact, AMPK-dependent phosphorylation of PGC-1α is required for the induction of PGC-1α promoter and for the transcription of many AMPK target genes (Jäger et al., 2007). Summarizing, both calcium-dependent pathways and AMPK modulate the activity and the expression of PGC-1α (Wu et al., 2002; Jäger et al., 2007). Therefore, many of the beneficial effects of exercise converge on the activation of PGC-1 signaling which is central for the mitochondrial and metabolic adaptation to exercise.

### Autophagy and ROS production

Different reports demonstrated that acute as well chronic exercise stimulates autophagy in different tissues (Grumati et al., 2011; He et al., 2012a,b; Lo Verso et al., 2014). Experiments conducted in inducible Atg7-deficient mice unraveled the mechanisms behind autophagy induction following exercise (Lo Verso et al., 2014). Interestingly, autophagy impairment does not affect exercise performance suggesting that autophagy is not required to sustain muscle contraction during exercise training (Lira et al., 2013; Lo Verso et al., 2014). However, autophagy is critical for muscle homeostasis after eccentric contractions. In fact, autophagy is necessary to remove dysfunctional mitochondria that have been altered by exercise in order to prevent excessive ROS production (Lo Verso et al., 2014). It is well known that exercise training and muscle contraction cause an increase in ROS production and a transient oxidative stress (Davies et al., 1982; Powers and Jackson, 2008). Indeed, oxidative stress has an important role in muscle signaling (Finkel, 1998; Rhee et al., 2000; Sena and Chandel, 2012), but can also induce damage to contractile proteins and organelles (Carnio et al., 2014). Although counterintuitive, antioxidant treatment leads to exercise intolerance in wild-type animals and do not ameliorate the physical performance of autophagy-deficient mice (Lo Verso et al., 2014). Moreover, control animals treated with antioxidants display mitochondrial dysfunction and autophagy impairment. Different reports support a crucial role of oxidative stress signaling during exercise in the maintenance of mitochondrial fitness (Gomez-Cabrera et al., 2005, 2008) and in the induction of molecular regulators of insulin sensitivity and antioxidant defense (Ristow et al., 2009). All together these results are consistent with the mitohormesis concept (Ristow and Schmeisser, 2014). Mitohormesis, the non-linear response to mitochondrial ROS formation, exhibits a dual dose-response (Ristow and Schmeisser, 2014). Accordingly, high ROS levels are detrimental due to the induction of oxidative stress with cellular damage. On the contrary, low ROS levels are essential for maintenance of cellular function as well as for improving oxidative stress for the promotion of health and lifespan (Ristow and Schmeisser, 2011; Owusu-Ansah et al., 2013). Therefore, understanding how mitohormesis is regulated could promise a potential application in the promotion of cellular homeostasis and longevity.

### IGF1/PI3K/AKT/mTOR pathway

Although both endurance as well as resistance exercise can promote significant health benefits, the molecular response signature will be different according to the frequency, intensity, duration and the type of exercise (resistance or endurance). The objectives of resistance exercise are an increase in muscle mass, strength and power. The resistance exercise-induced adaptive hypertrophic response is mediated by the anabolic factor insulin growth factor 1 (IGF1) via the IGF1/PI3K/AKT/mTOR pathway. Indeed, increased IGF1 levels precedes muscle hypertrophy achieved by functional overload (Adams and Haddad, 1996). Moreover, IGF1 stimulates fatty acid uptake and insuling sensitivity (Clemmons, 2012) explaining in part some of the beneficial effects of exercise. ATP citrate lyase (ACL) is a cytosolic enzyme that catalyzes the conversion of mitochondria-derived citrate into oxaloacetate and Acetyl-CoA important for lipid synthesis and acetylation. ACL activity is controlled by a post-translational modification via the IGF1/PI3K/AKT pathway. Overexpression of ACL in muscles improved mitochondrial function via stabilization of respiratory chain supercomplexes formation due to increases in cardiolipin synthesis (Das et al., 2015). Thus, IGF1 treatment of myotubes significantly increases mitochondrial respiration due to IGF1-dependent ACL activation suggesting that the activation of the IGF1-ACL pathway increases protein synthesis as well as stimulating the formation of the ATP necessary for this anabolic process (Das et al., 2015). The correlation of mitochondrial fitness to muscle homeostasis is confirmed by the fact that ACL overexpression in human myotubes is sufficient to induce hypertrophy while ACL activity is downregulated in sarcopenic and in dexamethasone-induced atrophic muscles (Das et al., 2015). All together, these different models offer a useful platform to further investigate the molecular mechanisms explaining how improvements in mitochondrial function can be translated into an increase in protein synthesis and therefore in hypertrophy.

## How does mitochondrial dysfunction activate muscle proteolytic pathways?

Muscle atrophy is an active process, controlled by specific signaling pathways and transcriptional programs (Lecker et al., 2004; Sandri, 2013). Of note, the muscle wasting transcriptome signature is characterized by the coordinated reduction of genes encoding key enzymes for ATP production and glycolysis (Lecker et al., 2004). Accordingly, alterations in mitochondrial morphology and function are frequently associated with atrophying muscles in aging (see Section “The Role of Disrupted Mitochondria Quality Control Pathways in Aging Sarcopenia”) as well as in several wasting conditions such as burn injury (Porter et al., 2013), intensive care unit-acquired weakness (Friedrich et al., 2015), insulin resistance (Crescenzo et al., 2014), chronic obstructive pulmonary disease (COPD) (Mathur et al., 2014), cancer cachexia (Antunes et al., 2014), and in different neuromuscular disorders (Katsetos et al., 2013). Over the last years, several *in vivo* genetic perturbation models indicate a key role of mitochondrial morphology and mitochondrial dysfunction signaling in the activation of nuclear programs controlling muscle loss. The maintenance of a functional mitochondrial network is indeed particularly important for tissues that are highly structured and metabolically active such as neurons, cardiac and skeletal muscles. These tissues are constituted by post-mitotic cells that do not divide and consequently, cannot dilute damaged/dysfunctional mitochondria through cellular division. Therefore, post-mitotic tissues depend on the activation of mitochondria quality control pathways to preserve or restore mitochondrial function.

**Figure 2:**
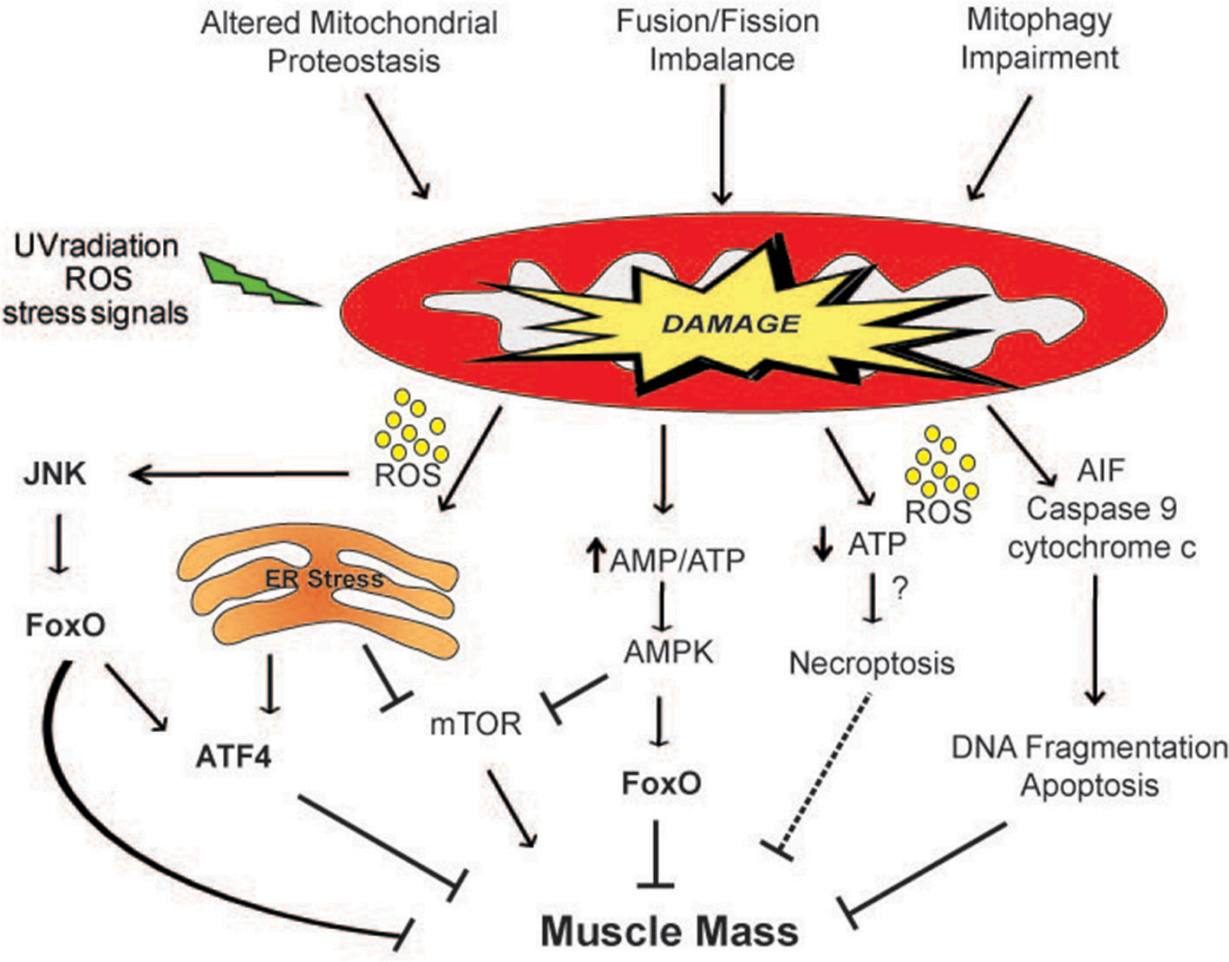
**Mitochondria-derived signaling pathways controlling muscle mass**. Mitochondrial network accumulates damage when the mitochondrial quality control mechanisms are impaired. ROS produced by the defective organelles induces muscle atrophy through the activation of the JNK-FoxO signaling pathway and of ER stress. Moreover, the release of dangerous factors such as AIF, cytochrome c and ROS can induce apoptosis and/or necroptosis. The activation of the energy sensor AMPK by an increase in the AMP/ATP ratio inhibits mTOR and directly phosphorylates FoxO3 (Ser413 and Ser588) increasing its transcriptional activity, affecting muscle mass. The dashed line indicates a mechanism that needs more studies.

Mitochondrial damage is attenuated with different defensive strategies including the activation of mitochondrial proteolytic systems, mitochondrial dynamics and the lysosomal degradation of mitochondrial cargo. For this reason, a failure in any of these systems predisposes to tissue dysfunction and degeneration (Figure 2). The importance of the actions of mitochondrial proteases, which remove misfolded or damaged mitochondrial proteins, in the control of muscle mass was confirmed by data obtained with several knockout animal models. Loss in the activities of the proteases PARL (Cipolat et al., 2006), Omi/HtrA2 (Jones et al., 2003; Martins et al., 2004), and Hax1 (Chao et al., 2008) lead to a similar phenotype, characterized by mitochondrial dysfunction, increased apoptosis, neurodegeneration and atrophy. A fine equilibrium of mitochondrial fusion/fission processes is needed to preserve muscle mass and prevent muscle wasting. This balance is regulated according to the physiological needs of cells.

### Fusion machinery and atrophy

Fused mitochondria are preferred when optimal mitochondrial function is needed. Indeed intermixing of mitochondrial content by mitochondrial fusion increases the bioenergetic capacity of the cell and is essential for the excitacion-contraction coupling in skeletal muscles (Eisner et al., 2014). Moreover, under conditions of high energy demand, mitochondrial fusion has a survival function, by protecting mitochondria against mitophagy, optimizing ATP production and leading to a sustained cell viability during nutrient deprivation (Gomes et al., 2011; Rambold et al., 2011). The biological importance of mitochondrial fusion in early development is revealed by the embryonic lethality of mouse knockouts for Opa1, Mfn1, and Mfn2 (Chen et al., 2003; Davies et al., 2007). However, few conditional tissue-specific knockout mice have been generated (Chen et al., 2011; Zhang et al., 2011) and some of them show a lethal phenotype therefore, limiting the studies of the role of these factors in mitochondrial function in adulthood. Therefore, little is known about the contribution of fusion machinery to mitochondrial function and tissue homeostasis in most tissues *in vivo*, including skeletal muscle fibers. The neurodegenerative disorders dominant optic atrophy (DOA) (Amati-Bonneau et al., 2008) and Charcot-Marie-Tooth type 2A (CMT2A) (Zuchner et al., 2004), are associated with loss of function mutations of Opa1 and Mfn2 genes, respectively. Noteworthy, patients affected with both DOA and CMT2A develop myopathies (Yu-Wai-Man et al., 2010; Feely et al., 2011). Likewise, neuromuscular defects related to precocious age-dependent axonal and myelin degeneration are present in mouse models resembling the Opa1 mutations most frequently associated with DOA patients (Alavi et al., 2009; Sarzi et al., 2012). Skeletal muscle from these models have alterations at the ultrastructural level with increased smaller mitochondria. In addition, mitochondria display disorganized cristae and accumulate lipid droplets (Sarzi et al., 2012).

#### Opa1 and muscle mass

Further support in the role of Opa1 in the homeostatic control of muscle mass comes from the observations obtained with a genetic model of controlled Opa1overexpression (Civiletto et al., 2015; Varanita et al., 2015). Opa1 transgenic mice are protected from acute muscle loss induced by denervation (Varanita et al., 2015) as well as from chronic muscle loss in a model of myopathy caused by muscle-specific deletion of the mitochondrial subunit COX15 (Civiletto et al., 2015). The analysis of the expression levels of the major atrogenes belonging to the ubiquitin-proteasome and of the autophagy-lysosome systems revealed a reduction of MuRF1 and LC3 expression levels in denervated Opa1 transgenic muscles. Furthermore, increased levels of Opa1 during denervation were sufficient to blunt denervation-induced mitochondrial dysfunction (Varanita et al., 2015).

#### Mfn2 and muscle loss

In line with the relevance of mitochondrial fusion proteins in the normal physiology of skeletal muscles, Mfn2 downregulation induces changes in ER-mitochondria communication that alters mitochondrial calcium buffering (Sebastian et al., 2012; Ainbinder et al., 2015). Moreover, Mfn2 protein levels are reduced in several catabolic conditions (Bach et al., 2005; Hernandez-Alvarez et al., 2010; Lokireddy et al., 2012). Mul1 is a FoxO-dependent ubiquitin-ligase involved in the ubiquitination and further degradation of Mfn2 during muscle atrophy. Accordingly, the maintenance of high Mfn2 proteins levels in starved muscles achieved by the downregulation of Mul1 partially protected from starvation-induced muscle loss (Lokireddy et al., 2012). On the contrary, the ablation of Mfn1 and Mfn2 in muscle-specific knockout mice induces a profound muscle atrophy. These models display mitochondrial dysfunction and compensatory mitochondrial proliferation, reduction of mtDNA as well as accumulation of point mutations and deletions of the mitochondrial genome (Chen et al., 2010). Mechanistically, Mfn2 deficiency in muscles induces a remodeling of the mitochondrial network leading to mitochondrial dysfunction and ROS production (Sebastian et al., 2012). Oxidative stress signaling is sufficient to activate an UPR that blunts insulin signaling via JNK and leads to insulin resistance (Sebastian et al., 2012). Mitochondrial fission is an essential component of the mitochondrial quality control mechanism. When mitochondria divide, two organelles are generated, one with high membrane potential that might undergo fusion and another fragment with low mitochondrial potential and reduced levels of OPA1 that will not rejoin the mitochondrial network and will be likely removed by autophagy (Twig et al., 2008).

### Fission machinery and atrophy

Impairment of fission leads to the disruption of the selective mitochondrial autophagic degradation followed by accumulation of dysfunctional organelles (Twig et al., 2008; Dagda et al., 2009; Kageyama et al., 2014; Ikeda et al., 2015; Song et al., 2015). In fact, mutations in mitochondrial fission components can be lethal. An infant girl born with a dominant-negative mutation of Drp1 presented alterations in brain development and in metabolism that cause neonatal lethality (Waterham et al., 2007). Moreover, genetic perturbations leading to the ablation of Drp1 in heart or brain are lethal (Ishihara et al., 2009; Wakabayashi et al., 2009; Kageyama et al., 2014; Ikeda et al., 2015; Song et al., 2015). Three different reports investigate Drp1 role in heart physiology. Disrupted mitochondrial fission, obtained with cardiac-specific ablation of Drp1, induces accumulation of defective mitochondria due to impaired autophagy/mitophagy, that over-time promotes cardiomyocyte death (Kageyama et al., 2014; Ikeda et al., 2015; Song et al., 2015). In addition, abnormalities in mitochondrial fission has also been implicated in neurodegenerative disorders such as Alzheimer's, Huntington, and Parkinson (Chaturvedi and Flint Beal, 2013). In skeletal muscle, atrophying conditions such as fasting, denervation or the overexpression of a constitutively active form of FoxO3 are characterized by alterations of the mitochondrial network (Romanello et al., 2010). Furthermore, the disruption of the mitochondrial network is a crucial amplificatory loop for the muscle atrophy program. The acute overexpression in muscles of the fission machinery *per se* promotes changes of mitochondrial morphology which are followed by mitochondrial dysfunction, energy stress and AMPK activation. As a consequence, AMPK modulates FoxO3 transcriptional activity in order to induce muscle atrophy via the activation of ubiquitin-proteasome and autophagy pathways (Romanello et al., 2010). Accordingly, inhibition of fission machineries or downregulation of AMPK is sufficient to protect against muscle loss in atrophying conditions (Romanello et al., 2010). These findings pave the way to further investigate the causal link between changes in mitochondria morphology and alterations in muscle homeostasis. A recent report investigates the consequences of mitochondrial network remodeling on muscle growth. The constitutive muscle-specific overexpression of Drp1 (Touvier et al., 2015) leads to muscle mass loss and decreased exercise performance (Touvier et al., 2015). In this mouse model, stress-induced mitochondria-dependent signals activate both, the UPR^mt^ and the eIF2α-ATF4-Fgf21 axis causing a reduction in protein synthesis and a blockade of growth hormones actions that prevent muscle growth (Touvier et al., 2015).

### Autophagy and atrophy

Mitophagy is a housekeeping mechanism important to keep under control the turnover of mitochondria in both, the basal state as well as under stress conditions. A finely tuned autophagic system is required for muscle maintenance. Notably, genetic manipulations to activate or inhibit autophagy results into muscle atrophy (Mammucari et al., 2007; Masiero et al., 2009; Romanello et al., 2010). Therefore, autophagy needs to be properly regulated, otherwise it can become detrimental instead of being protective. Several lines of evidence demonstrate that constitutive basal autophagy, which preserves mitochondrial function, is crucial for skeletal muscle homeostasis. Indeed, dysregulation of autophagic flux is detrimental for myofiber health and is a common feature of a group of muscle disorders with alterations of lysosomal proteins such as Danon or Pompe Disease as well as in the Vici Syndrome (Sandri et al., 2013). Moreover, defective autophagy plays a role in congenital muscular dystrophies caused by defects in collagen VI production (Grumati et al., 2010), laminin A/C (Ramos et al., 2012), or dystrophin (De Palma et al., 2012). These dystrophic models have in common a hyperactivation of Akt/mTOR signaling pathway that inhibits autophagy. Dystrophic muscles present accumulation of structurally altered mitochondria together with myofiber degeneration. Noteworthy, reactivation of autophagy flux by dietary or pharmacological tools, like rapamycin, cyclosporine A, or AICAR rescues the dystrophic phenotype by clearing the abnormal mitochondria (Grumati et al., 2010; De Palma et al., 2012; Pauly et al., 2012; Ramos et al., 2012). In agreement, suppression of autophagy by muscle-specific ablation of Atg5 and Atg7, the E3 ubiquitin ligases necessary for autophagosome formation, exacerbates fasting- and denervation- induced atrophy (Masiero et al., 2009). Moreover, impairment of autophagy induces accumulation of abnormal mitochondria, induction of oxidative stress, apoptosis, muscle atrophy, weakness and several features of myopathy (Raben et al., 2008; Masiero et al., 2009). Likewise, reduction of mitophagy caused by ablation of Pink and Parkin induces mitochondrial dysfunction and increased sensitivity to oxidative stress followed by muscle degeneration (Clark et al., 2006; Park et al., 2006; Billia et al., 2011). On the contrary, autophagic flux is increased in muscles in both exercise (Grumati et al., 2011; Lo Verso et al., 2014) and catabolic conditions (Sandri, 2013). For example, in skeletal muscle, mitochondrial dysfunction caused by the transient overexpression of FoxO3, Mul1 (Lokireddy et al., 2012), Bnip3, or Nix (Romanello et al., 2010) triggers autophagy and induces muscle atrophy (Mammucari et al., 2007; Romanello et al., 2010). Naf-1, the nutrient-deprivation autophagy- factor 1, is a ER-Bcl2 interacting protein identified as a novel autophagy regulator. Its downregulation is accompanied by the presence of abnormal mitochondria and increased autophagy that leads to muscle weakness and atrophy (Chang et al., 2012).

#### Mitophagy impairment and ROS production

Oxidative stress is increased when autophagy is dysregulated. ROS has detrimental effects both within and outside mitochondria. Indeed, autophagy deficient muscle show an increase of myosin and actin oxidation that reduces their sliding properties and causes muscle weakness. Anti-oxidant treatment restores a normal force generation and sliding properties even if it does not rescue muscle atrophy (Carnio et al., 2014). Mutations of the Superoxide Dismutase (SOD1) gene is behind 20% of cases of inherited amyotrophic lateral sclerosis (ALS). ALS is a neurodegenerative disease characterized by motoneuron degeneration and muscle atrophy. Aberrant ROS production affects mitochondrial function producing a feedback loop that in turn exacerbates mitochondrial damage. Transgenic muscle-specific mice expressing the SOD1 G93A mutation, an ALS-associated human SOD1 mutation display muscle atrophy which is mainly through autophagy activation (Dobrowolny et al., 2008).

#### Autophagy and muscle metabolism

Skeletal muscle plays a significant role in the metabolic control of glucose, lipids, and energy. Importantly, these processes are coordinated with changes in mitochondrial content, shape, and/or function. Therefore, the question now arises as to how modulation of the mitochondrial quality pathways and specifically of mitophagy can affect muscle metabolism. Despite conflicting results, two different studies investigate the connection between autophagy/mitophagy and metabolic regulation in muscles. The first one, uses transgenic mice with knock-in mutations on Bcl2 phosphorylation sites that cannot trigger autophagy by fasting or exercise. These mice show decreased exercise endurance, impaired exercise-induced glucose uptake and are more sensitive to high fat diet-induced glucose intolerance (He et al., 2012a). Since the transgene is expressed in all cell types, it cannot be ruled out whether the observed effects are resulting from the combination of the actions exerted by different metabolically active tissues (liver, heart, muscle, brain). In the second study, muscle-specific autophagy deficient mice (Atg7 knockout) are protected from high-fat diet induced obesity and insulin resistance. The protective effects depend on dysfunctional mitochondrial signals sufficient to activate an UPR via ATF4, which induces Fgf21 expression (Kim et al., 2013). Therefore, it is not clear how autophagy can fine-tune the metabolic pathways in response to different cellular cues. An important future goal is to establish the metabolic impact of mitochondrial quality control pathways in skeletal muscle.

### Oxidative stress and atrophy

As already discussed, ROS act in a hormetic fashion (Ristow and Schmeisser, 2014). The beneficial or detrimental action will depend on the level and persistance of ROS flow, as well as on the antioxidant capacity of target cells (Barbieri and Sestili, 2012). While low levels of ROS are associated with positive effects on muscle physiology, on the other hand, excessive production of free radicals accelerates muscle proteolysis (Powers et al., 2012; Reid et al., 2014). Accordingly, the exposure of oxidants to myotubes results in muscle atrophy (McClung et al., 2009; Gilliam et al., 2012). In addition, several models of disuse atrophy like mechanical ventilation, limb immobilization, hindlimb unloading or bed rest are associated with increased ROS production (Pellegrino et al., 2011). Recent evidence indicates mitochondrial ROS production as a required signaling step to induce mitochondrial dysfunction and muscle disuse atrophy (Min et al., 2011; Powers et al., 2011; Talbert et al., 2013b). Mechanistically, oxidative stress can impinge on proteolytic pathways in multiple ways; First, ROS can regulate NF-kB and FoxO transcriptional factors (Dodd et al., 2010) activating the autophagy-lysosome and the ubiquitin-proteasome systems (Taillandier et al., 1996; Levine et al., 2008, 2011; Andrianjafiniony et al., 2010; Hussain et al., 2010; Brocca et al., 2012). Second, the muscle proteases calpain (Taillandier et al., 1996; McClung et al., 2009; Andrianjafiniony et al., 2010; Nelson et al., 2012; Talbert et al., 2013a) and caspase-3 (Levine et al., 2008; Nelson et al., 2012; Talbert et al., 2013a) are activated during disuse atrophy. Third, oxidized proteins are more prone to proteolysis because: (a) their susceptibility to be degraded by caspase-3 and calpain is enhanced, in part because of unfolding of the proteins (Smuder et al., 2010), (b) they can be directly degraded by the proteasome without being ubiquitinated (Grune et al., 2003), and (c) ubiquitin conjugation activity is increased by oxidative stress (Shang et al., 1997).

Summarizing, all these data strongly support the involvement of mitochondrial morphology and/or function in the activation of signaling pathways that control muscle mass.

## The role of disrupted mitochondria quality control pathways in aging sarcopenia

Sarcopenia is defined as the progressive age-related decline in muscle mass and force. It is considered a primary risk for developing major human pathologies that can result into disability and increased vulnerability to death. It has been estimated that roughly, 1.1 and 1.9/kg of muscle mass are lost per decade in women and men, respectively (Janssen et al., 2000). Of note, muscle strength precedes muscle loss, declining three-times faster than muscle loss (Goodpaster et al., 2006). Age-associated muscle loss begins at a slow rate around 30 years of age, then it accelerates and starts to be noticeable after 45 years of age (Janssen et al., 2000). Beyond the sixth decade of life more severe alterations such as spinal motor neurons alterations can be observed (Booth et al., 1994) while the most affected age is over 80 years old (Cruz-Jentoft et al., 2010). The average life expectancy of human beings is rapidly increasing. According to the National Institute on Aging (NIH), in 2010 8% of the world's population were people over 65 years old and this number is expected to double by 2050. Although the resulting clinical, economical and social relevance of sarcopenia, the mechanisms that drive the age-related loss of muscle mass and function are not completely understood. Sarcopenia is a complex multifactorial process. Recently, nine primary hallmarks of aging were specified (Lopez-Otin et al., 2013). Among these, mitochondrial dysfunction has been long suggested as one of the mechanisms of aging sarcopenia (Trounce et al., 1989; Melov et al., 2007; Gouspillou et al., 2010, 2014a,b; Picard et al., 2011; Carnio et al., 2014).

### Decreased mitochondrial biogenesis

It has been shown that muscle mitochondrial content declines with age (Conley et al., 2000; Short et al., 2005; Chabi et al., 2008), however, there is no clear consensus (Mathieu-Costello et al., 2005; Callahan et al., 2014; Gouspillou et al., 2014b). Mitochondrial biogenesis is important for determining mitochondrial content and function. It depends on the transcription, translation and import of new proteins into pre-existing organelles. In agreement to the observed age-associated mitochondrial content reduction and dysfunction, PGC1-α mRNA and protein levels are reduced in aged muscles (Short et al., 2005; Chabi et al., 2008; Safdar et al., 2010; Ghosh et al., 2011; Joseph et al., 2012; Ibebunjo et al., 2013). On the other hand, muscle-specific PGC1-α transgenic mice are protected from the reduction in mitochondrial function and content observed during aging (Wenz et al., 2009). Moreover, overexpression of PGC1-α is sufficient to prevent age-related muscle loss (Wenz et al., 2009). Blunted mitochondrial biogenesis in aging is supported also by the fact that nascent mitochondrial precursors are more prone to be degraded by cytosolic factors (Huang et al., 2010).

### Age-associated mitochondrial oxidative damage

Several mechanisms necessary for mitochondrial homeostasis are dysregulated in sarcopenia. In fact, during aging, ROS production increases over a certain threshold, overcoming cellular antioxidant defense. Therefore, the initial homeostatic response of ROS signaling is subverted and accelerates the age-associated damage (Lopez-Otin et al., 2013). The resulting oxidative stress, induces post-translational modifications which compromise protein function (Baraibar et al., 2013). The target proteins modified by oxidative stress (the oxy-proteome components) in aged muscles have been identified recently, by the resolution of carbonylated proteins (an indicator of oxidized damaged proteins) by two-dimensional gel electrophoresis (Lourenco Dos Santos et al., 2015). Many of them are mitochondrial proteins which affect mitochondrial proteostasis (Lourenco Dos Santos et al., 2015; Ross et al., 2015).

### Mitochondrial dynamics changes

Despite certain discrepancy between reports, the balance between fusion and fission is altered in sarcopenic muscles. Some studies reported that muscle mitochondria in old rats are smaller than young controls (Ljubicic et al., 2009; Iqbal et al., 2013). On the other hand, giant mitochondria were observed in aged muscles of houseflies (Rockstein and Bhatnagar, 1965), muscle cells of humans (Beregi et al., 1988), mice (Beregi et al., 1988), and rats (Navratil et al., 2008). Analysis of the longitudinal vs. transversal mitochondrial orientation with transmission electron microscopy reveals that muscle mitochondria from aged mice are much more enlarged and branched than young muscles (Leduc-Gaudet et al., 2015). Even though the authors did not find any difference in the expression of the dynamic machineries between young and old mice, they suggest that the alteration of mitochondrial morphology in old muscles is the result of a fusion/fission imbalance toward mitochondrial fusion (Bori et al., 2012; Leduc-Gaudet et al., 2015). In contrast, other groups find a downregulation only of Opa1 (Navratil et al., 2008; Joseph et al., 2012) or an upregulation of the fission machinery (Iqbal et al., 2013). Interestingly, a transcriptomic and proteomic study found that reduced expression of all the components of the mitochondria dynamic machineries correlates with age-related muscle loss in rats (Ibebunjo et al., 2013). The employment of new tools, like FIB-SEM, would be useful to correlate mitochondrial dynamics alterations with mitochondrial function in sarcopenic muscles.

### The role of mitophagy in sarcopenia

Several evidences indicate an alteration in mitochondrial turnover during sarcopenia. Considering the decline of mitochondrial biogenesis together with the progressive accumulation of macromolecules and dysfunctional organelles it seems to create a likely basis for impaired mitochondrial function and removal during aging. In agreement, some autophagic regulators like LC3 lipidation, Atg7, p62, beclin1, Bnip3, Parkin, and LAMP2 decrease with age (Russ et al., 2012; Joseph et al., 2013; Carnio et al., 2014; Gouspillou et al., 2014b). Moreover, a genetic muscle-specific model of autophagy deletion (Atg7 knockout mice) displays precocious aging characterized by increased oxidative stress, mitochondrial dysfunction, muscle loss and weakness and degeneration of neuromuscular junctions. All together, these results put mitophagy central in age-related mitochondrial dysfunction and critical to prevent age-related denervation and weakness. In contrast, others found increased levels of lipidated LC3 (Wenz et al., 2009), Atg7, beclin1 (Fry et al., 2013), parkin, and p62 (O'Leary et al., 2013). The divergence in these results highlight the necessity of complementing the analysis of the expression of single autophagic regulators with assays to evaluate the complete autophagic process by flux measurements for the correct interpretation of the results (Klionsky et al., 2012).

### Interventions to attenuate mitochondrial dysfunction in aging

Even though, aging sarcopenia is an unavoidable consequence of getting old, it can be reduced with regular exercise and with nutritional interventions. Notably, the benefits of both strategies are mediated by activation of autophagy. Exercise training increases mitochondrial content and turnover by activating both, mitochondrial biogenesis (Little et al., 2011) and autophagy (Vainshtein et al., 2014). As mitochondrial turnover and function are both altered in sarcopenic muscles, exercise seems to be a good countermeasure to improve mitochondrial function (Jubrias et al., 2001; Short et al., 2003; Menshikova et al., 2006; Melov et al., 2007) and therefore, to reduce the sarcopenic process (Jubrias et al., 2001; Melov et al., 2007; Kern et al., 2014; Zampieri et al., 2015). Interestingly, Melov et al. (2007) defined the aging transcriptome signature. The sarcopenic muscle profile was enriched with genes associated with mitochondrial dysfunction compared with young muscles (Melov et al., 2007). Genes important for energy production and mitochondrial function decreased with age. Examples of these genes are the genes encoding the ubiquinol-cytochrome C reductase hinge protein (UQCRH), the β subunit of succinyl-CoA synthase (SUCLA2), and the C subunit of succinate dehydrogenase (SDH). Six months of resistance training were sufficient to revert the transcriptome expression profile of the genes associated with mitochondrial metabolism and electron transport to the expression levels characteristic of young people. However, at the functional/phenotypical levels resistance exercise partially improved muscle strength (Melov et al., 2007). Importantly, the benefits of exercise in reverting the sarcopenic phenotype do not have the same effect on advanced age (over 80 years of age) which have limited muscle plasticity (Slivka et al., 2008; Raue et al., 2009), with the oldest ones being the most affected by the consequences of aging muscle atrophy (Cruz-Jentoft et al., 2010). Caloric restriction (CR) is a powerful nutritional intervention that has been reported to increase lifespan, to maintain metabolism at a more youthful-like state and to prevent chronic diseases (Fontana and Partridge, 2015). The investigation of the effects of lifelong CR in rats muscles demonstrated that CR increases the expression of some autophagic proteins like LC3, Atg7, and 9 and LAMP2 attenuating the age-dependent decline in autophagy. Therefore, the chronic activation of autophagy decreases oxidative damage as well as apopoptotic DNA fragmentation and is sufficient to reduce age-related myocyte degeneration maintaining skeletal muscle homeostasis (Wohlgemuth et al., 2010). Chronic moderate CR adaptation in muscles from humans (30% lower energy intake) and rats is mediated by three major pathways: IGF1/insulin/FoxO, mitochondrial biogenesis, and inflammation (Mercken et al., 2013). CR-mediated responses lead to reduction of IGF1/AKT pathway with the consequent increase of FoxO3 and 4 transcript as well as an upregulation of FoxO-dependent antioxidant system and autophagy-related genes (Mercken et al., 2013). Moreover, CR induces a transcriptional reprogramming profile of molecular pathways that become more similar to the profile of young individuals (Mercken et al., 2013). The involvement of age-dependent mitochondrial decline in sarcopenia as well as the physiological significance of this decline clearly needs more studies. In years to come it will be exciting to see if manipulations aimed at enhancing mitochondria quality control pathways will be sufficient to promote a healthy aging of muscles.

## Final considerations

In the last years our understanding of the different mechanisms controlling mitochondrial morphology/function and the impact of these pathways in the homeostatic control of muscle mass has experienced an unprecedented advance. However, there are still many challenges that should be addressed. First, since the components of the mitochondria quality control system are closely interconnected, the alteration in one system can impinge on the activation/inhibition of the other repair mechanism. Therefore, this interplay, which is of fundamental importance for the integration of mitochondrial function within the network, should be considered in future studies. Second, mitochondria are social organelles that establish direct or indirect (via mitochondria-derived vesicles) contacts with other cellular components. Indeed, mitochondrial communication with the endoplasmic reticulum, peroxisomes, and lysosomes/vacuoles are hot topics of study at the moment. Then, it will be of great interest to investigate the functional consequences of these interactions in the physiology of muscle in the future. Finally, mitochondria-derived metabolites can shuttle within the cell and within the whole organism. Thus, exploring the interrelation between mitochondria quality control pathways and the metabolic regulation of muscle mass as well as the muscle-interorgans crosstalk should give additional insights to understand the way these relationships can be manipulated to treat human diseases.

## Author contributions

VR and MS wrote the manuscript.

### Conflict of interest statement

The authors declare that the research was conducted in the absence of any commercial or financial relationships that could be construed as a potential conflict of interest.
